# Qualitative research within trials: developing a standard operating procedure for a clinical trials unit

**DOI:** 10.1186/1745-6215-14-S1-P98

**Published:** 2013-11-29

**Authors:** Frances Rapport, Clare Clement

**Affiliations:** 1 Swansea University, Swansea, UK

## Background

Qualitative research methods are increasingly used within trials to address broader research questions than quantitative methods can address alone. Qualitative methods enable health professionals, service users and other stakeholders to contribute their views and experiences when evaluating health care treatments, interventions or policies. They can influence trial design, allowing for a fuller engagement with research questions, aims and objectives and clarify the complexities of people's social relationships, their experiences of healthcare and the context of clinical research.

## Aim

To develop a qualitative methods Standard Operating Procedure (SOP) which would allow the value and role of qualitative methods as part trials to be recognised, and appropriately incorporated within trials.

## Method

Health researchers, including trialists, clinicians and qualitative researchers, worked collaboratively to develop a qualitative methods SOP for the West Wales Organisation for Rigorous Trials in Health (WWORTH).

## Results

The Qualitative Methods SOP developed defines good practice in designing and implementing qualitative components of trials, presents the role of qualitative research while allowing flexibility in method and process. Its basic principles include: qualitative researchers should contribute from the commencement of trial with qualitative potential; the qualitative component should have clear aims and objectives; and the main study publication should report on the qualitative component alongside other aspects.

## Conclusion

We recommend that CTUs consider developing a qualitative methods SOP to enhance the conduct of qualitative trials including qualitative data collection, analysis and outputs. This could improve the value and visibility of the role of qualitative methods, in order to enhance clinical trials.

